# Stem Cells: A Historical Review about Biological, Religious, and Ethical Issues

**DOI:** 10.1155/2021/9978837

**Published:** 2021-04-29

**Authors:** Ioannis Alexandros Charitos, Andrea Ballini, Stefania Cantore, Mariarosaria Boccellino, Marina Di Domenico, Elisa Borsani, Riccardo Nocini, Michele Di Cosola, Luigi Santacroce, Lucrezia Bottalico

**Affiliations:** ^1^Interdepartmental Research Center for Pre-Latin, Latin and Oriental Rights and Culture Studies (CEDICLO), University of Bari, Bari, Italy; ^2^Department of Emergency and Urgency, National Poisoning Centre, Riuniti University Hospital of Foggia, Foggia, Italy; ^3^Department of Biosciences, Biotechnologies and Biopharmaceutics, Campus Universitario “Ernesto Quagliariello”, University of Bari “Aldo Moro”, Bari, Italy; ^4^Department of Precision Medicine, University of Campania “Luigi Vanvitelli”, Naples, Italy; ^5^Department of Interdisciplinary Medicine, University of Bari, School of Medicine, Bari, Italy; ^6^Division of Anatomy and Physiopathology, Department of Clinical and Experimental Sciences, University of Brescia, Brescia, Italy; ^7^Section of Ear Nose and Throat (ENT), Department of Surgical Sciences, Dentistry, Gynecology and Pediatric, University of Verona, Verona, Italy; ^8^Department of Clinical and Experimental Medicine, University of Foggia, Foggia, Italy

## Abstract

Stem cells can be used to replace damaged cells or regenerate organs and have broadened our knowledge of the development and progression of certain diseases. Despite significant advances in understanding stem cell biology, several problems limit their use. These problems are related not only to the growth of tumors in animal models and their rejection in transplant cases but also to ethical and social issues about the use of embryonic cells. The ethical-scientific debate on this type of cells has taken on great interest both for their application in regenerative medicine and for the potential possibilities in the field of cell and gene therapy. Different points of view often have the expression of a perception that depends on scientific goals or opportunities or on religious traditions and beliefs. Therefore, as the questions and doubts about when life begins, so do the answers for the use of these cells as therapy or otherwise. So, in addition to the origin of stem cells, there are currently some social bioethical (such as political and legislative issues) and religious dilemmas. The purpose of the study is aimed at being a narrative on the history of stem cells and the evolution of their use to date, as well as to clarify the bioethical position of the various religions today in comparison with the social ones regarding the research and use of embryonic and adult ones. Hence, their biological hypostasis regarding the concepts of “conception” and “fertilization” and their development and therapeutic use compared to those of the main theological doctrines.

## 1. Introduction

Stem cells are undifferentiated cells that have not yet developed structures or proteins and are characteristic of a specific type of cell or tissue. Thus, they contribute to the creation of all mature cells in the human body and are the basis of every cell, tissue, and organ. A stem cell is able to guarantee both self-renewal and differentiation. The way to do this is through its asymmetrical division. However, cell division can be both symmetrical and asymmetrical. The symmetrical division produces identical cells (cell cloning). Asymmetric division is typical of stem cells and therefore creates cells destined to differentiate (different from the cell of origin) together with cells identical to those of origin (i.e., still stem cells). In this way, self-renewal is guaranteed, and at the same time, a progeny of differentiated cells is created which in turn will be transferred to the affected tissues or organs [1, 2]. Thus, the stem cells must also be able to differentiate into the various types of cells and to regenerate functional tissues in vivo. Stem cells are a population of undifferentiated cells characterized by (I) their ability to proliferate widely (ability to self-renew) and to differentiate into different types of cells and tissues (potential for differentiation) and (II) usually arise from a single cell (clonal property). There are five types of stem cells according to their origin: (a) embryonic stem cells (ESCs) are isolated from the internal cell mass of the blastocyst. They consist of populations of pluripotent cells that can produce the primitive ectoderm during embryogenesis. A particular group of stem cells is those of the dental-derived stem cells (d-DSCs) which can differentiate *in vitro* into layer-like tissues of mesoderm, endoderm, and ectoderm such as those of adipocytes and neural cells [2–6]. (b) Amniotic epithelial cells (AECs) arise from the amniotic membrane of the human placenta. They express multivalent ESC markers (such as oct-4, Nanog, and alkaline phosphatase), do not express telomerase, and do not form teratomas *in vivo* after transplantation. They can differ *in vitro* from both endoderm (such as pancreatic endocrine cells and hepatocytes), mesoderm (such as cardiomyocytes), and from the ectoderm (such as in nerve cells) [4, 5]. (c) Fetal stem cells (FSCs) are mostly isolated (from embryonic cadaver tissue organs or are from tissue-specific embryonic stem cells), up to the 12^th^ week of pregnancy. They can therefore be transplanted without rejection reactions and have the therapeutic advantage over ESCs of not causing teratomas *in vivo* [7–10]. (d) The umbilical cord epithelium (UCE) is derived from the epithelial amniotic membrane and is a source of pluripotent stem cells. They can differentiate into various functional progenitor cells (such as hematopoietic, dendritic, neural, hepatocytes, pancreatic cells, and endothelial cells), and (e) adult somatic stem cells are produced during ontogenesis [11–13]. They are found in specialized areas in almost all mammalian organs and tissues such as bone marrow, heart, kidneys, brain, skin, eyes, gastrointestinal tract, liver, pancreas, lungs, breasts, prostate, testicles, and ovaries. Moreover, four types of multipotent adult stem cells have been described: (1) isolated from long-term cultures of nonphenotypic adherent cells from human or mouse/rat mesenchymal tissue such as bone marrow and muscle tissue, (2) unrestricted ones isolated from umbilical cord blood (UCSCs) cultures, (3) inducible (MIAMI) isolated from marrow, and (4) multipotent from human bone marrow (h-BMSC). However, there is a simpler distinction of stem cells which is the one that divides them according to their origin and their developmental plasticity that is in embryonic and somatic. However, there are multiplicative somatic stem cells (Table 1) [11, 13, 14].

**Figure 1:**
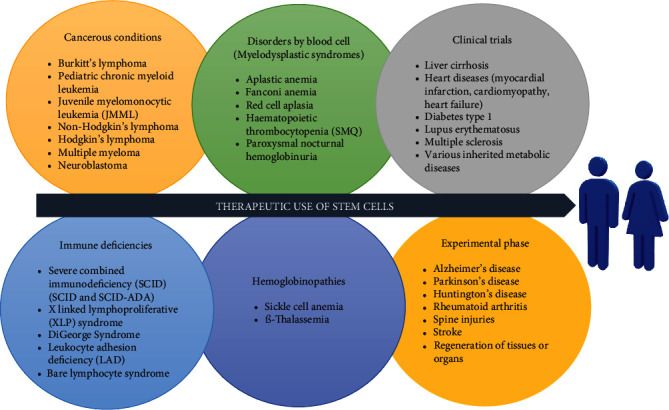
Summary of the diseases treated or even therapies in clinical trials or experimentation with stem cells.

Stem cells are used in the research field of medicine for the treatment of various diseases such as hematological (e.g., bone marrow transplantation), ophthalmology (e.g., age-related macular degeneration), endocrinological (diabetes), neurological, genetically modified fat cells, and drug development (Figure 1).

**Figure 2:**
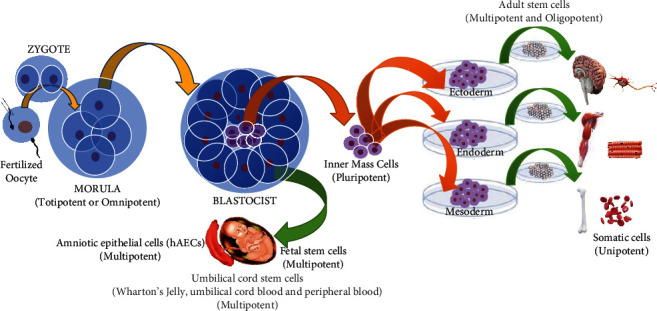
The origin, the collection process, and the plasticity of stem cells: after fertilization of the natural oocyte or in vitro the pluripotent embryonic stem cells are created as internal cell mass inside a blastocyst. The stem cells show that their range of potential is wide and that cells of one tissue can, under experimental conditions and with appropriate culture cell, reprogram to mature cells in another, different tissue from which they come from. So, they can function as multiples. This is called plasticity and is the basis of cell therapy. Fetal stem cells are found within the organs of the fetus. To this source belongs the embryonic corpse tissue (which can be obtained after a spontaneous abortion due to illness, etc.) and afterwards with the appropriate culture, reprogrammed and functioning such as multipotent cells.

To date, three stem cell-based therapies have been considered: (a) already differentiated cell transplantation with specific types of cells that derive from embryonic or somatic stem cells and include patient's own stem cells and grow and differentiate under special processing conditions in the laboratory (e.g., insulin-producing cells for the treatment of diabetes), (b) stimulation of endogenous stem cells in an individual (e.g., by administering growth factors) with the possibility of inducing or increasing the processes of self-repair, and (c) direct administration of stem cells to the patient, so that a colonization process begins in the desired part of the body and thus the differentiation continues into the desired cell type (Figure 2) [15–18].

However, depending on the choice of technique and the type of stem cells, we will also have certain limitations (Table 2). Hematopoietic stem cell transplantation from bone marrow, peripheral blood, or cord blood is used to treat hematological malignancies or congenital immunodeficiencies (such as *β*-thalassemia, Fanconi anemia, sickle cell anemia, acute and chronic leukemia, F leukemia, non-Hodgkin's lymphoma, Hodgkin's disease, and others). Stem cell transplantation belonging to the same patient (autologous) was also used to preserve bone marrow in patients who had received a high dose of chemotherapy. The use of h-BMSCs as a therapy to modulate immune responses is known. In fact, it has been shown that these cells can express histamine H1, H2, and H4 receptors and histamine itself then stimulating the production of interleukin 6 (IL-6) [18–20]. Finally, several studies mention the role of stem cells in the relationship with periodontitis antibodies and particularly in CVD (cardiovascular disease) biomarkers in the early stages. It was noted that some patients with periodontitis had serum and salivary increase in *Aggregatibacter actinomycetemcomitans* high IgG titers and high hs-CRP (high-sensitivity C-reactive protein) with a negative effect. The burden of periodontal bacteria can lead to an increase in plasma and salivary levels of suPAR (soluble urokinase-type plasminogen activator receptor) and hs-CRP [21–23]. In fact, suPAR proved to be a valuable prognostic biomarker for the correlation between periodontitis and related cardiovascular diseases [21]. It has recently been noted that during a periodontal disease that is associated with endothelial dysfunction, there is a relevant relationship between the disease and the EPCs (circulating endothelial progenitor cells) [24]. EPCs are a subtype of bone marrow-derived stem cells, and they have hematopoietic and endothelial stem cells and thus participate in the integrity of the vessels. Indeed, EPC has been suggested as an indicator of endothelial dysfunction and the cumulative risk of cardiovascular disorders. EPC identification occurs through the search for markers CD34, CD133, and the kinase insertion domain receptor (KDR). Furthermore, EPCs increase is correlated with the presence of positive to serum biomarkers [21–24].

Autologous transplantation is also used for the treatment of difficult autoimmune diseases but also as a means of gene therapy which is still in the experimental stage. Additionally, stem cell transplantation is used as a treatment for some types of cancer (such as breast and neuroblastoma). As for the treatment of diabetes, perhaps the production of functional pancreatic cells from stem cells ready for transplantation could be a new therapeutic approach [25, 26].

Scientific interest has mostly turned to embryonic stem cells as in the case of certain neurological diseases (e.g., stroke, Alzheimer's disease, and Parkinson's disease). On this prospect, the use of neural stem cells (NSCs) as regenerative therapies, the combination with the cannabinoids for medical use is very promising. It has been noted that they act as potent regulators of NSCs, thus, also having the potential to modulate various neurogenic characteristics. Using embryonic stem cells, certain types of nerve cells can be produced ex vivo, which, if transplanted into the body, could repair focal organic damage (such as experimentally, even in spinal injuries). Furthermore, using the differentiation power of stem cells, congenital defects such as *Krabbe disease*, cheiloschisis, osteogenesis imperfecta, and others can be cured [25–29]. In the dental field, dental mesenchymal cell (MSCs) research can be applied for the replacement of dental and oral tissues against dental caries, periodontitis, etc., as well as the use of platelet-rich plasma (PRP) and platelet-rich fibrin (PRP) extracts for the dental implantology process [13, 30–34].

Finally, for treatment in patients with inflammatory bowel disease (IBD), they develop microbiota dysbiosis with immune dysregulation. Furthermore, it was noted that mesenchymal stem cells (MSCs) can produce similar effects, and targeted investigations on the interactions between these cells and gut microbiota could lead to a new therapeutic approach. The use of probiotics and/or fecal transplantation can be an effective adjuvant therapeutic strategy [35–39].

## 2. Stem Cells in the History

### 2.1. The Early Years

The two zoologists Theodor Heinrich Boveri (1862-1915) and Valentin Häcker (1864-1927) used the term stem cell to describe cells committed to give rise to the germline. Boveri was also a comparative anatomist who during his cytology and genetic studies discovered that some cells could regenerate with subsequent functional differentiation. On this basis, he thinks that even cancer cells start from a cell with chromosome scrambled leading to continuous and uncontrollably dividing cell [40]. He thus wanted to explain the theory of embryonic nature tumor etiology and pathogenesis as previously interpreted by Julius Cohnheim as did Max Askanazy (1865-1940), Felix Marchand (1846-1928), and Robert Bonnet (1851-1921) with the blastomere theory of teratoma-like tumors. However, still today, some oncogenetic theories for some tumors still concern the cells of this type of cells [41–49]. The histologist Franz Ernst Christian Neumann who was carrying out his studies on bone marrow and Alexander Alexandrowitsch Maximow (1874-1928) claim that there is a common cell precursor that differs to give all the mature blood cells. On this Maximow, he developed the concept of polyblasts. It will be later that these cells with the aim of regenerating and differentiating themselves were called stem cells by Ernst Haeckel (1834-1919). He is starting from the Mendelian concept thinking that could not always explain heredity and phenotypic traits and so he puts into action the new concept of research that he will call phenogenetics. In fact, he argued that in multicellular beings, there is a first line of cells that call *Stammzellen (Stamm*: phylum + *zellen*: cells) which leads to phylogeny that is to evolution through evolution proliferation and differentiation capacity. Then, he introduced the foundations for the theory of hematopoiesis [50].

In the early 60s, Ernest Armstrong McCulloch (1926-2011), a biophysicist, and James Edgar Till, (born 1931) a cell biologist, were the pioneers for the study of stem cells with the quantitative clonal method. After they had introduced the cells into the bone marrow of the previously irradiated laboratory mice, they observed nodules at the level of the spleen. In fact, these cell colonies each came from a single progenitor cell [51]. Later together with the molecular biologist Lou Siminovitch (born in 1920), they realize that cells could functionally self-renew by creating these colonies [52]. Thanks to the oncologist and immunologist Georges Mathé (1922-2010) in 1958, we have the first successful allogeneic bone marrow transplant with people who are not relatives, and in 1963, he treated a patient with leukemia for the first time thanks to the bone marrow transplant. Previously, together with Marcel Legrain and René Kuss, he performed the first kidney grafts among unrelated donors with success [53].

### 2.2. Towards the Evolution of the 21st Century Era

In 1981, the two biologists Sir Martin John Evans (born in 1941) with Matthew Kaufman (1942-2013) cultivated mouse embryonic stem cells for the first time in the laboratory, and subsequently in 2007, together with Mario Capecchi and Oliver Smithies, they won the Nobel Prize in physiology and medicine. In 1988, the first umbilical cord blood stem cell transplant took place in a child with Fanconi's anemia. Since then, more than 6,000 transplants have been performed in which the transplant is entrusted to a relative or another stem cell recipient, provided there is compatibility (heterologous transplant) [54].

This is because they used embryonic stem cells to induce specific gene modifications in mice by isolating the embryonic stem cell from the embryo and implanting it in adult female mice. This will open new horizons for research and therapy. In 1992, the first public and private stem cell bank was established in the United States. In 1995, umbilical cord stem cells were introduced of equal value in therapeutic applications with bone marrow stem cells [54].

In 1998, the biologist James Alexander Thomson through his research led him to the discovery of human embryonic stem cells (published under the title Embryonic Stem Cell Lines derived from Human Blastocysts), and in 2007, he discovered the method of human-induced pluripotent stem cells (iPS), i.e., by converting skin cells into cells that closely resemble human embryonic stem cells (published article under the title Induced Pluripotent Stem Cell Lines Derived from Human Somatic Cells) [55, 56].

In 2001, the first study on umbilical cord stem cell transplantation in adults was published, and in 2004, Gesine Koegler and colleagues found that in the umbilical cord blood, in addition to hematopoietic stem cells, there are pluripotent stem cells [57].

Finally, in 2012, Shinya Yamanaka and John Gurdon were awarded the Nobel Prize for Physiology or Medicine for the discovery that mature cells can be converted to stem cells; hence, they can be manipulated to become pluripotent [58].

## 3. Ethical Issues in the Use of Stem Cells

### 3.1. Epistemology and Ethical Social Approach

Stem cell research then is linked to moral issues that are raised around the world. On the one hand, the development of adequate and innovative therapies for the treatment of serious diseases makes their research indispensable, but on the other, moral questions emerge from the use of embryos and therefore the limits and conditions of this research. In addition, there is the question of the limitations of intellectual property on the use of research materials and how to access medical and therapeutic technologies. It is important to understand the future use of gametes, embryos, or somatic cells offered by donors together with the protection of privacy (including that of donors). Hence, using them raises questions relating to personal data [59]. At the same time, there is a debate on the safety and efficacy of treatments in progress, as well as on the understanding, by patients, of the origin of the materials used in the treatments. For stem cells isolated from the umbilical cord, there are two main related issues: (a) the appropriate time that donor consent should be obtained for the use of the resulting medical data and (b) the related issues concerning maintenance and the cold storage in specific banks. Concerning bone marrow mesenchymal stem cells, questions arise regarding pain and risks to the donor during the cell isolation process [60].

Then, questions are raised relating to complete information consent of the participants on the whole experimental process. The importance of some institutional issues concerns the careful consideration of the intellectual property right that governs stem cell research related to medical treatments and ethics. In fact, for this reason, the guidelines have been created over time by the International Society for Stem Cell Research (ISSCR) [17, 61]. Thus, it is important to review the objectives of the information and consent process on cell and tissue donors and to collect data thereafter. Finally, the collection of other somatic cells (such as adult skin cells) raises some distinct problems such as limited discomfort and the patient's physical risk during the isolation process. In fact, the first source of stem cell production is the excess fertilized eggs left over from *in vitro* fertilization, and that after the removal of the pluripotent stem cells, the fertilized eggs are destroyed [62]. This is where two moral issues arise: (a) that it is intolerable to annihilate (“kill”) fertilized eggs that can potentially develop into a human being; (b) morally, it is unacceptable to use these “potential” human beings to an end. However, according to medical and biological science, the fertilized egg of the first fourteen days does not yet contain a personalized life, as it begins to form after fourteen days of fertilization [63]. Therefore, there is no existence of a “human person” that can be attributed to the fetus in the first few days. It is not subject to duties and rights to be able to recognize a situation like that attributed as in the human being. Thus, it is not about “killing” by taking stem cells.

Furthermore, there can be no concern when there are abortion laws that allow the destruction of the fetus until the first twelve weeks of pregnancy since the freedom of the woman is valued by the legislator as a superior legal good and by the fetus of the first three months. Thus, a possible source of stem cells is cadaveric embryonic tissue after spontaneous or voluntary abortion. But for this to happen, it is necessary to obtain the consent of the pregnant woman if this would not lead to an insult to the woman's personality [64]. Therefore, you must be adequately informed before giving your consent for both the use of the fetal tissue and the source of research funding [59]. However, the creation of human embryos for research purposes only is prohibited.

The distinction between pluripotent and omnipotent stem cells is seen as ethically crucial in medicine's science. After the complete development of the pluripotent stem cells, there is the possible evolution of them towards complete and equal organisms such as the embryo of origin [65, 66]. Thus, it is likely that a reproductive technique may be developed in the future which will create people with similar external characteristics. This is morally at fault because the creation of human beings would weigh on the free development of the personality and on their autonomy of life. This cannot happen in a society that respects the value of human life. Some bioethics committee proposes that it would be more ethically appropriate to strictly prohibit the acquisition of stem cells for the purpose of culturing omnipotent. Therefore, to obtain stem cells from fertilized eggs, it is necessary to obtain the written consent of the gamete donors, as these fertilized eggs are elements of their personality. This consent must specify that the fetus will not be used for third party reproductive purposes and that in case of refusal it will not have negative consequences on future medical care needs. However, research on fetal tissue is indicated in case of interruption of medical problems as it can help to understand the causes of the anomalies with prenatal or preimplantation tests when it comes to couples who have resorted to *in vitro* fertilization. Thus, thanks to the bioethical dilemmas regarding the extraction of stem cells from the human embryonic blastocyst, an attempt was made to extract the stem cells three days after fertilization in the division phase of 6-8 cells after biopsy and without altering the development of the embryo which is then implanted in the uterus [67].

As regards adult stem cells, this source does not present legal but bioethical problems. This is because it does not endanger the person from whom they are removed, and for this reason, scientists should focus on finding these same stem cells. The condition to be considered is the donor's consent with the guarantees already provided in detail in the convention on human rights and biomedicine. As in previous stem cell sources, the financial counterpart agreement is being strictly prohibited. It would also be advisable to keep the donor anonymous as in transplants. Another source of stem cells would be that of cloning. Cloning as a process is the replacement of the egg nucleus with the nucleus of a mature somatic cell, which contains all the genetic information necessary to create a human organism.

Nevertheless, reproductive cloning is explicitly prohibited [68]. Therefore, without allowing for an evolution of this organism but only taking the stem cells in the first days of its evolution, we can define therapeutic cloning; therefore, the reception of stem cells by organisms created by intentional cloning and for the sole purpose of research is prohibited. Therapeutic cloning should be exempted from the general prohibition in Article 18 of the Council of Europe Convention on Human Rights and Biomedicine [69]. The storage and management of stem cells are important. However, another aspect of the stem cell issue then is the function of the banks that store and prepare these cells for use. Precisely because the use of stem cells as tools for disease treatment is limited, the existence of such banks has no real value.

However, if these banks exist and operate, it is imperative that those interested in their services are protected and not misinformed. Interested parties should be informed about medical data relating to stem cell research and about the law on the protection of personal data [67]. Finally, the new pandemic disease from the novel SARS-CoV-2 coronavirus and the planned procedures that perform allogeneic and autologous hematopoietic cell transplantation (HCT) have been disrupted. The European Society for Blood and Marrow Transplantation (EBMT) has drawn up guidelines for the more comfortable management issues of transplant candidates for both recipients and donors [70–72].

### 3.2. Theological Bioethical Approach Dilemmas on the Use of Stem Cells

According to the Christian's doctrine only Human, such as a psychosomatic unit, from the moment of conception, can become free thanks to his/her own will by the grace of God, in communion, that is, with divine energy, thus, removing his body from tenderness and from decomposition. From the moment of its conception, the fetus, as a “potential person,” is a structured psychosomatic entity. The main approach to the question of the beginning of life is made by the Christian thought, with spiritual criteria refers to a human being who was created as an image of the creator God and exists as an independent being from the first moment of his conception. After these considerations, we understand that the Christian point of view is different from medical epistemology. This is because, only at the beginning of a pregnancy is the concept of “potential human being” introduced, and at birth, one can speak of a human face, since from that moment, the newborn is entitled to special legal protection. On the other hand, “fertilized eggs” or *in vitro* embryos, which are placed in a nutrient medium, will not grow with certainty. In fact, some may not even reach the blastocyst stage or may not be of sufficient quality to be transferred to the female uterus. Instead, the valid and grown ones must be cryopreserved after the fifth day in case they are redundant because otherwise, they will stop growing and therefore existing. Continuing this previous thought, we can understand that this “image” is sometimes attributed to the autonomy of man and sometimes to the soul together with the body [73–75].

The Roman Catholic Church, represented by the Bishop or Pope of Rome, is against the use of embryonic stem cells from embryos created exclusively for therapy and research (this appears at the Pontifical Academy for Life in August 2000), which concludes with the use of only stem cells by adults for this purpose. Indeed, there is the suggestion of the use of adult stem cells and that this type of cell is more suitable for the advancement of research and therapeutic applications [73, 75, 76]. The position of the Roman Catholic Church, from 1869 onwards, is that the moment of conception is also the beginning of the entity of the human person. However, the Roman Catholic Church, before 1869, which by decision of Pope Pius IX, adopted now of conception, as the beginning of the life of a complete man, according to Thomas Aquinas. He claimed that the fetus acquires a soul after 40 days of fertilization. So, during its development, it is not considered human until it acquires a soul, and it can happen with a continuing pregnancy. At the beginning, the embryo lives like a plant because it is not an independent essence. After the transformation from the state of a vegetable being into an animal and that only in the final phase of its development is the rational soul created (which is given only by God) transforming it into the human essence. Thus, the Catholic ethical doctrine does not allow the use of embryonic stem cells from embryos created exclusively for this purpose, by means of assisted reproduction procedures or for the purpose of reproductive cloning [77, 78].

The point of view of the Protestant doctrines on the stem cell issue presents several points of view. Mainly, the theological and ethical point of view begins with the doctrine that recognizes human beings as entities that will have a relationship first with God and then with others and with itself. Therefore, the human fetus is considered a developing human being that, if not born, will not have these relationships. Thus, he opposes the use of early embryonic stem cells, as well as the question of reproductive cloning, because all this is considered against human dignity [73, 74, 78]. Finally, the Lutheran theologian and professor of systematic theology at Pacific Lutheran Theological Seminary (USA), Theodore Frank Peters, have very moderate ideas. He criticizes the Catholic doctrine for having combined the concept of the divine union of the soul with the body (that has become from the animal in Human rational essence) with the uniqueness of the new genome until it is deposited on the walls of the uterus between the 12th and the 14th day later fertilization. He also makes considerations about the excess and abandoned embryos that are placed for cryopreservation in the refrigerators of assisted reproduction clinics; in fact, since they are destined to be destroyed, he thinks whether they should be attributed to research. Their use for research purposes can lead to important therapeutic breakthroughs [79, 80].

In the Orthodox church (former imperial Christian Roman church) doctrine, represented by the Ecumenical Patriarch and Archbishop of New Rome or Constantinople (today Istanbul), the research on the use of embryonic stem cells is based on the position on the moral status of embryos. According to this theological doctrine, the psyche (*ψυχή*) is an immaterial divine gift that builds the essence of a personality at the origin of its life. Through being it completely differentiates itself from the inanimate and unreasonable creation, summarizing within it (as being with intellect) the creation both material and spiritual (divine) that causes the life. The body is initially presented in the form of a single cell, the fertilized egg, resulting from the fusion of the nuclei of two germ cells, a male and a female, which through this karyogamy phenomenon unite both the living energies of the souls of the carriers giving new vital energy, i.e., the psyche. So, it already preexists from the beginning immediately after this merger. As the body develops, the psyche also reveals its energies [81].

Therefore, without psyche, there can be not existing life, because in biblical theology it is considered as the “breath of life.” For every scientific process, the Orthodox Church carefully follows the reasoning and methods that can go to the detriment of the concepts of the Human “*prosopon*” (“face”: as the distinct divine individual energy) and the Human *hypostasis* (the individual unique existence) to which they confer a unique and supreme value in the rational beings thus the humans [82].

Hence, the fetus has the status of a perfect human being, to be subsequently perfected by divine energy. The Orthodox Church considers that God during the creation of the human being gave him all the necessary conditions to develop his abilities to exercise for good. This means that man has acquired a specific divine quality and every scientific achievement is a consequence of this. However, the Church recognizes the therapies that come from somatic stem cells, but also expresses her theological objections when derived from embryonic cells [80]. These concepts determine what is morally acceptable or not in the Orthodox Church as a fundamental bioethical principle. This theological and bioethical axis has remained unchanged over time in Orthodox theology compared to other Western Churches. However, the theological bioethical theory of the Orthodox Church refuses to search for stem cells or part of a variety of embryonic stem cells but has a positive effect on migratory cell search of adult stem cells. Finally, it allows the research and use of umbilical cord stem cells, because they come from an adult organism at the time of delivery and their collection does not present an ethical or moral theological dilemma [77–80].

According to the Jewish religion, the fetus according to the Old Testament and in the Talmud after the forty days of its development is considered in part a human being, which has respect and protection. Research on embryonic stem cells is morally acceptable since the embryo before 40 days is “as if it were water” and not an entity with a real soul.

For the Islamic religion, referring to the Koran, the fetus acquires psyche and image at the end of its fourth month of pregnancy. He has since been considered a person who enjoys rights. Hence, stem cell research may be acceptable if stem cell research is acceptable because the fetus cannot be considered a complete human being [65, 75, 78, 79].

In Buddhism, it is particularly important that the use of embryonic stem cells can be used if one intends to do research aimed at healing and improving the lives of patients, but research done for financial gain is not acceptable. In Hinduism, however, the destruction of embryos is morally not permissible. However, has no official position on stem cell research. The issue in Buddhism is that it does not accept any cause of pain in all living beings but considering that before 14 days the fetus does not feel pain, we can say that research on embryonic stem cells can be performed (Table 3) [65, 75, 78, 83].

## 4. Conclusions

The use of stem cells in the treatment of diseases is one of the major research achievements of recent years. Isolating fetal and adult stem cells is challenging. Umbilical cord blood stem cells are less of a challenge to isolate as they are obtained from placental blood at birth after the umbilical cord is cut. In recent years, adult stem cells can be reprogrammed back to their pluripotent state. This is an important evolution in this scientific field. The use of embryonic stem cells is controversial because it requires the destruction of an embryo, and adult stem cells are preferred for the treatment of diseases. When the stem cells come from the recipient himself, there is no risk of rejection. The potential of stem cells is varied as the stem cells of one tissue can, under appropriate conditions, mature into cells of another different tissue and function as omnipotent and not simply as multipotent. This phenomenon is called plasticity and is the basis of cell therapies. Regenerative medicine is the one that could benefit most from the use of pluripotent stem cells. Activating pluripotent stem cells to produce specialized cell types will enable a renewable source of cells and tissues to be used as transplants and alleviate many diseases and disabilities. Thousands of patients around the world have already benefited from stem cell treatment that is still evolving today for many important pathologies such as neurological ones. Thus, the use of stem cells contributes to (a) basic research on human development, (b) the safe and more specialized development of drugs, and (c) the treatment of repair or replacement of damaged tissues and organs of the body in the context of the regenerative medicine field. The embryonic stem cell or the embryo *in vitro* could be used for scientific purposes only because the fate of its survival simply depends solely on its implantation in the uterus. On the other hand, there is the opinion that even the embryo *in vitro* has a potential that is its ability to evolve into a human being and, therefore, considers every stem cell precious for the same reason. For science and in particular, for biologists and doctors, the fetus is a set of cells without a “substantial” human being and that “therapeutic” cloning differs from “reproductive” cloning because it aims at curing diseases and therefore must be morally acceptable.

In summary, the process of obtaining human embryonic stem cells from embryos *in vitro* finds the opposite side of the Christian Churches, because the embryo becomes a person from the first moment of conception. But here, we can notice in an interdisciplinary way, that is, between theology and science a different interpretation of terms that leads to a concurrent error. It is obvious that the perceptions and objections that exist today in the main Christian doctrines that are both in the Catholic and Evangelical and Orthodox Churches seem to be based on a misunderstanding of what is it, a conception whether made natural or with medical assistance. Therefore, one cannot speak of an early fetal existence because it does not exist.

The *in vitro* fertilized egg, although it is in a nutrient medium and can grow under suitable conditions (not, obviously in all cases) to the blastocyst stage, does not constitute an “embryo”. To do this, it is necessary to transfer it to the uterus and to grow after successful “implantation.” So, there is conception (both in the case of *in vitro* fertilization and through the natural process) only if the fertilized egg is successfully implanted and the pregnancy begins. Otherwise, there is no conception. Thus, there exists an error interpretation between the term conception and fertilization, and the use of the term “embryo” as the fertilized egg (or the embryo *in vitro*), which cannot yet be considered as a real fetus but only if transferred to the uterus for its subsequent development. Hence, the definition of the term conception should not be equated with the term fertilization. Therefore, it cannot be considered that an early fetal existence exists, even the egg fertilized *in vitro* up to the blastocyst stage, does not constitute an “embryo”. Because, as we have already mentioned, its transfer, successful implantation, and thus its growth in the uterus are mandatory. Finally, it is of fundamental importance to start a constructive debate on the issue that allows the comparison and ethical supervision of embryonic stem cells. Due to the recent embryonic stem cell research, there is still no clear and specific regulation for each case. The 2016 ISSCR guidelines offer a reason for comparison and therefore for updating given the latest knowledge and experiments.

## Figures and Tables

**Table 1:** The different sources of stem cells with different plasticity and differentiation.

Stem cell plasticity				
Totipotent/omnipotent	Pluripotent	Multipotent	Oligopotent	Unipotent
They are cells with the most undifferentiated cell form during embryonic development (e.g., the fertilized oocyte (zygote)) up to the stage of the first blastomeres (i.e., three to four days after fertilization). Are single cells that can give rise to a new organism with adequate maternal support. So, it can give rise to all extra-embryonic tissues, tissues of the body, and of the germ line.	They can differentiate into cell types from the ectoderm, endoderm, and mesoderm, which then produce all cell types for all tissues and organs. The best-known pluripotent stem cells are embryonic (isolated from the internal cell mass (ICM) of the blastocyst).	They can differentiate into discrete cell types (e.g., several types of blood cell-like lymphocytes, monocytes, neutrophils, bone cells or other nonblood cell type, and others. The best now is the mesenchymal cells (MSCs) in the bone marrow, adipose tissue, ∗Wharton's jelly in umbilical cord blood, dental tissues, and peripheral blood.	These stem cells can self-renew and differentiate into two or more cells that belong to a specific type of tissue (e.g., hematopoietic stem cells, the bronchioalveolar stem cells, or BASCs).	They can self-renew and differentiate into a single specific cell type forming a single cell line (e.g., the muscle stem cells).

∗Inside the Wharton's jelly (substantia gelatinea funiculi umbilicalis) in umbilical cord, there is a cell population that has stem characteristics and is made up of the mesenchymal cells of the layer (MSCs). They exhibit cell adhesion characteristics while phenotypically expressing a specific set of surface antigens (including CD73, CD90, and CD105 stem cells).

**Table 2:** Main differences and limitations for use of stem cells.

Limits of stem cells		
Embryonic stem cells		Adult stem cells
In vitro fertilization	Nuclear transport	Production of a limited number of cell types. They are not found in all tissues. Difficulties in identification, isolation, preservation, and cultivation in the laboratory.
Limited number of cell lines for federally funded research needs. Risks for the creation of teratomas (tumors) from the transplantation of undifferentiated stem cells.	It has not yet been achieved in humans. Risks for the creation of teratomas (tumors) from the transplantation of undifferentiated stem cells.	

**Table 3:** Main bioethical concerns of stem cells.

Bioethical reserves of stem cell resources	
Embryonic stem cells	Adult stem cells
In vitro fertilization/somatic cell nuclear transfer (SCNT)∗	Nonsignificant bioethical reservations on their use, which mostly concern informed consent and noncommercialization
Consent of donors and subsequent destruction of blastocysts (social/religious questions) ∗The administration of oocytes requires the consent of the donor and suspicions for their possible use in reproductive cloning.	

## Data Availability

The data used to support the findings of this study are included within the article.
